# A high-accuracy, low-cost blood test for Alzheimer’s disease: validating P-tau181/Aβ42 in real-world cohorts

**DOI:** 10.3389/fnagi.2025.1599761

**Published:** 2025-06-16

**Authors:** Dequan Liu, Hang Li, Qing Liu, Haiyan Li, Yuanyuan Tang, Kaiting Cheng, Tong Li, Yulan Chu, Xiaodong Jia, Wenying Yu, Hailan Zhou, Keqiang Yan

**Affiliations:** ^1^Tianjin Kingmed Diagnostics Laboratory Co. Ltd., Tianjin, China; ^2^Tianjin Key Laboratory of Multi-omics Precision Diagnosis Technology for Neurological Diseases, Tianjin, China; ^3^GuangZhou Kingmed Diagnostics Laboratory Co. Ltd., Guangzhou, Guangdong, China

**Keywords:** Alzheimer’s disease, plasma biomarkers, P-tau181/Aβ42, SIMOA, flow cytometry

## Abstract

**Objective:**

To evaluate the diagnostic performance of plasma P-tau181/Aβ42 measured via flow cytometry as a cost-effective tool for Alzheimer’s disease (AD) diagnosis.

**Methods:**

A cohort study involved 123 healthy controls, 60 AD/mild cognitive impairment (MCI) patients, 34 subcortical ischemic vascular disease (SIVD) patients, and 34 frontotemporal dementia (FTD) patients. Plasma P-tau181 and Aβ42 levels were measured using flow cytometry and cross-validated with Single-molecule Array (SIMOA). Publicly available Chinese cohort data were reanalyzed for comparative performance.

**Results:**

The P-tau181/Aβ42 ratio revealed significant differences between groups. A reference interval (0–0.109) achieved 96.2% diagnostic accuracy (95.0% sensitivity, 96.7% specificity) for AD versus controls, distinguishing AD from SIVD (88.3% accuracy) and FTD (86.2% accuracy). Flow cytometry-based P-tau181/Aβ42 showed 88.3% consistency with SIMOA-based P-tau217, while SIMOA-based P-tau181/Aβ42 achieved 92.3% accuracy.

**Conclusion:**

Flow cytometry-based P-tau181/Aβ42 offers a cost-effective and accurate diagnostic method for AD, with performance comparable to SIMOA. This biomarker supports scalable AD screening in secondary healthcare settings, overcoming accessibility and cost barriers in resource-limited environments. This biomarker supports scalable AD screening in secondary healthcare settings, overcoming accessibility and cost barriers in resource-limited environments.

## Highlights

 • Flow cytometry-measured P-tau181/Aβ42 achieves 96.2% accuracy in distinguishing AD from controls.

 • P-tau181/Aβ42 differentiates AD from non-AD dementias (86%–88% accuracy), validated against SIMOA P-tau217.

 • Cost-effective flow cytometry enables scalable AD screening in resource-limited settings.

## Introduction

Alzheimer’s disease (AD), a chronic neurodegenerative disorder, represents a significant threat to global geriatric health. By 2019, approximately 50 million individuals worldwide were suffering from AD, a number projected to triple to 152 million by 2050 due to population aging (Ashton et al., 2022). This trend highlights AD’s status as a critical public health challenge, compounded by its escalating socioeconomic burden: global costs are projected to rise to 16.9 trillion by 2050 (Brookmeyer et al., 2007). AD progresses over 20–30 years, beginning with the asymptomatic preclinical phase, transitioning to mild cognitive impairment (MCI), and culminating in dementia. Recent therapeutic advancements in AD underscore the critical need for sensitive biomarkers to identify candidates for early intervention (Cai et al., 2023; Chong et al., 2021). However, in China, critical gaps continue to exist in AD diagnosis, as evidenced by low rates of early clinical engagement and predominant late-stage detection]95% presenting with moderate-to-severe stages at initial diagnosis (Fowler et al., 2022)]. Furthermore, misdiagnosis (30%) and underdiagnosis (> 60%) rates remain unacceptably high (Gauthier et al., 2021; Hansson, 2021), underscoring the necessity for accessible diagnostic tools.

Current diagnostic strategies, as recommended in China’s AD management guidelines, include clinical evaluations, imaging (Positron Emission Tomography/Magnetic Resonance Imaging, PET/MRI), and cerebrospinal fluid (CSF) biomarker analysis (Jack et al., 2010). Clinical assessments, though widely used, lack sensitivity and reliability, heavily relying on clinician expertise (Jack et al., 2010). Conventional neuropsychological tests detect cognitive decline only after several years of Aβ and tau pathology accumulation, rendering them inadequate for early diagnosis (Jack et al., 2024). Emerging digital cognitive tools show promise in identifying subclinical impairments but remain experimental and not scalable (Liang et al., 2016). Aβ-PET and Tau-PET imaging significantly enhance early detection by visualizing Aβ plaques and tau tangles *in vivo*. However, their prohibitively high costs and limited availability restrict widespread adoption (Nandi et al., 2022). CSF biomarkers (e.g., Aβ42, total tau, phosphorylated tau [p-tau]) provide direct evidence of neurodegeneration but require invasive lumbar punctures and specialized laboratories, limiting their practicality (Palmqvist et al., 2021). Blood-based biomarkers, enabled by ultra-sensitive platforms like single-molecule array (SIMOA), promise scalable screening (Rabinovici et al., 2019; Santangelo et al., 2019). The International AD Conference recently endorsed plasma P-tau217 as a PET-correlated diagnostic biomarker (Sims et al., 2023). Nevertheless, reliance on SIMOA’s proprietary technology imposes financial barriers, highlighting the need for cost-effective alternatives. The plasma P-tau181/Aβ42 ratio, reflecting CSF Aβ pathology (Snellman et al., 2023), correlates strongly with CSF P-tau181 levels in early AD (Tian et al., 2019). Here, we validate plasma P-tau181/Aβ42 via flow cytometry technology—a widely accessible platform—as a practical diagnostic tool. Our findings demonstrate its accuracy in differentiating AD from controls and non-AD dementias, offering a non-invasive, cost-effective alternative for scalable screening in resource-limited settings.

## Materials and methods

### Study design and participants

This study conducted a comprehensive analysis of data from two cohorts. Cohort I consisted of patients recruited from the Memory Clinic at Tianjin Medical University General Hospital, encompassing 60 cases of AD/MCI, 34 subcortical ischemic vascular disease (SIVD), and 34 frontotemporal dementia (FTD). These patients, aged between 44 and 82, were diagnosed by professional physicians using specific criteria. Specifically, AD patients met the International Working Group (IWG)-2 criteria and had positive Aβ PET results, SIVD patients met the diagnostic criteria for vascular dementia according to the International Society of Vascular Behavioral and Cognitive Disorders, and FTD patients met the revised Frontotemporal Dementia Consensus criteria for behavioral variant frontotemporal dementia or the classification recommendations for semantic variants or non-fluent variants of primary progressive aphasia. Additionally, this study enrolled 123 cognitively unimpaired healthy individuals from a clinical laboratory center in Northern China, Hainan General Hospital in Southeast China, and the Affiliated Hospital of Guizhou Medical University in Southwest China. These participants exhibited no subjective symptoms of cognitive decline, demonstrated normal neurological and neuropsychological examination results, had MMSE scores greater than 26, CDR scores of 0, and ranged in age from 21 to 85 years. The study received approval from the Medical Research Ethics Committee of the General Hospital of Tianjin Medical University (Ethics Number: IRB2023-YX-214-01). All participants provided written informed consent at recruitment. Cohort II originated from a study on AD within the Chinese population, conducted by Xuanwu Hospital (Cai et al., 2023). Utilizing the Simoa platform, the research aimed to detect levels of Aβ40, Aβ42, Tau, and P-tau181 in the plasma of 122 healthy controls and 126 individuals diagnosed with AD.

### Plasma biomarkers measurement

Blood samples were collected using EDTA anticoagulant tubes to obtain 5 mL of peripheral venous blood from each subject. The collected blood was centrifuged at 3,000 rpm for 5 min in a high-speed centrifuge to separate the plasma, which was then stored at −80°C for subsequent analysis. The plasma levels of Aβ42, Aβ40, and P-tau181 were measured using the BD Canto flow cytometer, following the protocols outlined in the Sakyi Biotech kit. The BD Canto flow cytometer is a widely used instrument for precise and reliable detection of biomarkers in plasma. Plasma P-tau217 levels were measured using the fully automated Simoa HD-X protein detection analyzer (Quanterix Corporation, USA), which employs Simoa technology to achieve highly sensitive and specific measurements. The choice of these specific instruments and kits was based on their proven reliability and accuracy in biomarker detection, ensuring robust and reproducible results.

### Statistical analysis

Normally distributed measurement data were presented as mean ± standard deviation and subjected to *t*-tests, while non-normally distributed measurement data were described using median and interquartile range and assessed through non-parametric tests of Wilcoxon tests. Referencing WS-T402-2012 “Establishment of Reference Intervals for Clinical Laboratory Tests,” in conjunction with practical clinical applications, an elevated level of P-tau181/Aβ42 is considered abnormal. Therefore, the 95th percentile (P95) is calculated to establish a one-sided reference interval. Meanwhile, coefficient of variation (CV) was used to assess the inter-assay reproducibility, with calculated formula as following: CV = (sd/mean)*100. Sensitivity and specificity analysis of discrimination was expressed by receiver operating characteristic (ROC) curve and area under the curve (AUC). Boxplots and points were used to present the distributions of original values of plasma biomarkers. All data were analyzed using R software.

## Results

### Reference interval and diagnostic performance of flow cytometry P-tau181/Aβ42

A total of 273 participants were initially enrolled, with 22 outliers excluded due to hemolysis or technical assay failures. The final cohort comprised 123 controls, 60 AD/MCI patients, 34 FTD cases, and 34 SIVD patients. The demographic and clinical features of all participants are presented in Table 1.

**TABLE 1:** Demographics and biomarker concentrations of all participants.

Characteristics	Control (*n* = 123)	AD/MCI (*n* = 60)	FTD (*n* = 34)	SIVD (*n* = 34)
Age	53.0 (33.0, 60.0)	69.0 (62.8, 73.2)	66.5 (63.0, 69.8)	70.0 (66.0, 73.8)
Gender, female (%)	78 (63.4%)	42 (70.0%)	20 (58.8%)	6 (17.6%)
MMSE score	28.0 (27.0, 29.0)	22.0 (17.0, 25.0)	21.0 (15.3, 24.5)	22.5 (19.0, 24.8)
P-tau181	0.563 (0.329, 0.770)	2.195 (1.902, 2.843)	0.861 (0.659, 1.101)	0.774 (0.528, 1.266)
Aβ42	11.085 (9.612, 13.787)	9.382 (6.340, 12.937)	9.757 (7.890, 11.271)	9.712 (8.067, 10.387)
P-tau181/Aβ42	0.050 (0.030, 0.070)	0.232 (0.169, 0.387)	0.090 (0.073, 0.134)	0.082 (0.058, 0.137)

**FIGURE 1:**
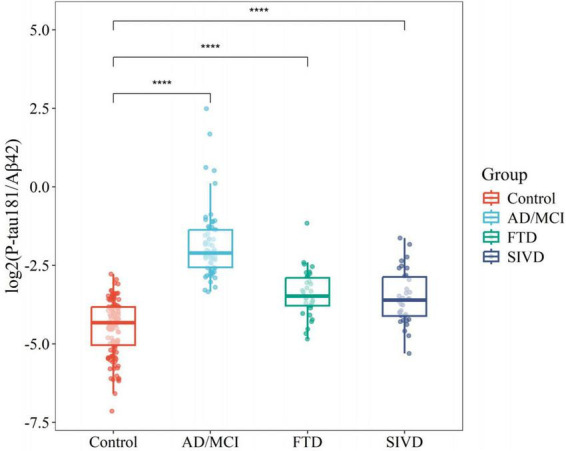
Boxplots comparing flow cytometry platform P-tau181/Aβ42 between different disease groups and controls. ****P < 0.0001.

**FIGURE 2:**
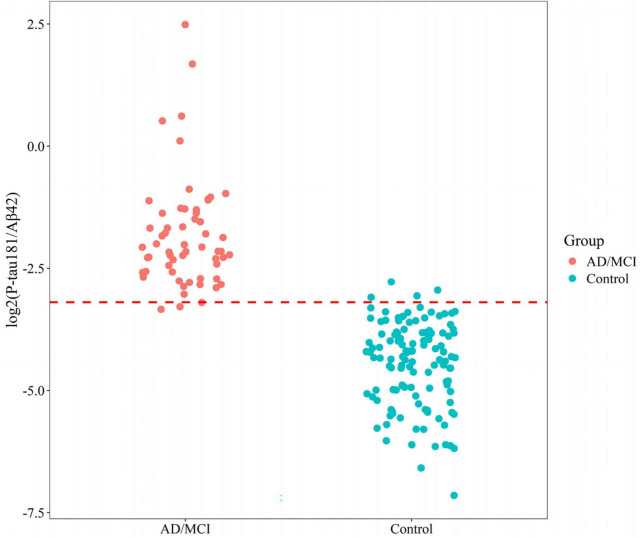
Reference interval distribution of P-tau181/Aβ42 in AD/MCI and control groups based on flow cytometry platform.

Relative to controls (median 0.050 [0.030–0.070]), plasma P-tau181/Aβ42 levels were significantly elevated in AD/MCI (median 0.232 [IQR 0.169–0.387]), FTD (0.090 [0.073–0.134]), and SIVD (0.082 [0.058–0.137], Figure 1). Besides, Generalized Additive Model (GAM) was used to assess between-group differences in P-tau181/Aβ42 levels adjusting for age and sex as covariates and the results supported the robustness of the reported group differences (Supplementary Table 1). This finding underscores the biomarker’s ability to differentiate between healthy individuals and those with degenerative diseases, highlighting its potential utility in distinguishing between control groups and neurological disorders. To establish a reference interval for the P-tau181/Aβ42 index among the general population, the interval was defined as 0–0.109 by calculating P95, as illustrated in Figure 2. At this threshold, P-tau181/Aβ42 demonstrated robust diagnostic performance in clinical settings for distinguishing between AD patients and healthy individuals, with accuracy of 96.2% (95% CI: 93.4%–99.0%), sensitivity of 95% (85.2%–98.7%), and specificity of 96.7% (91.4%–99.0%) (Table 2), despite suboptimal weak inter-assay reproducibility (CV < 52.8%).

**TABLE 2:** Diagnostic performance evaluation of flow cytometry platform P-tau181/Aβ42.

	AD vs. control	AD vs. SIVD	AD vs. FTD
Accuracy	0.962	0.883	0.862
Sensitivity	0.95	1	0.9
Specificity	0.967	0.676	0.794

### Differential diagnostic performance of P-tau181/Aβ42 across disease groups

**FIGURE 3:**
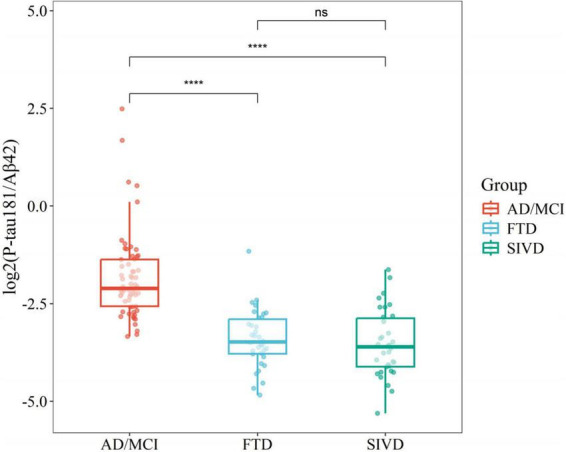
Boxplots comparing flow cytometry platform P-tau181/Aβ42 across disease groups. ****P < 0.0001.

Non-parametric Wilcoxon tests were employed to assess differences in P-tau181/Aβ42 levels across diagnostic categories, revealing statistically significant variations between AD and FTD [*p* < 0.05 or SIVD (*p* < 0.05)]. However, no significant difference was observed between FTD and SIVD (*p* > 0.05), highlighting the limited specificity of P-tau181/Aβ42 for non-AD dementias (Figure 3).

**FIGURE 4:**
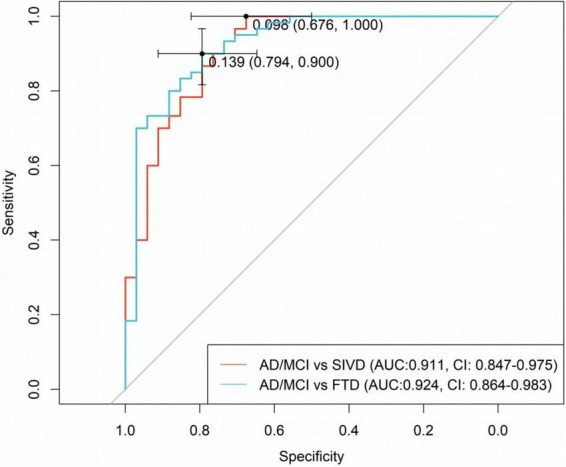
ROC analysis for AD/MCI vs. SIVD and AD/MCI vs. FTD.

To determine optimal cutoff values for diagnostic accuracy, ROC curve analysis was conducted. As shown in Figure 4, for distinguishing AD and SIVD, the plasma P-tau181/Aβ42 ratio demonstrated 100.0% sensitivity (95% CI: 100.0%–100.0%) for correctly classifying AD, with an AUC of 0.911 (95% CI: 0.847–0.975) at the optimal cutoff of 0.098. For distinguishing AD and FTD, the ratio achieved 90.0% sensitivity (95% CI: 81.7%–96.7%) and an AUC of 0.924 (95% CI: 0.864–0.983) at the cutoff of 0.139. These findings underscore the biomarker’s robust ability to differentiate AD from both healthy controls and non-AD dementias, thereby establishing its clinical relevance in diagnostic applications.

### Diagnostic concordance between flow cytometry P-tau181/Aβ42 and SIMOA P-tau217

P-tau217 detection utilizing the SIMOA platform has been established as a valuable diagnostic tool in the identification of AD. To assess the diagnostic concordance between P-tau181/Aβ42 and P-tau217, SIMOA P-tau217 detection was conducted on the samples. In a subset of participants (94; 38 AD/MCI, 56 controls), P-tau181/Aβ42 (flow cytometry) exhibited 88.3% overall concordance with SIMOA P-tau217 (Table 3), and the two methods showed relatively similar diagnostic performance (Supplementary Table 2). This finding suggests that the two biomarkers are mutually corroborative in the diagnosis of AD, underscoring the robust reliability of P-tau181/Aβ42 as a diagnostic biomarker for AD.

**TABLE 3:** Diagnostic consistency between flow cytometry platform P-tau181/Aβ42 and SIMOA platform P-tau217.

	P-tau181/Aβ42		
P-tau217		AD/MCI	Control
	AD/MCI	36	4
	Control	7	47

### SIMOA platform validation of P-tau181/Aβ42

**FIGURE 5:**
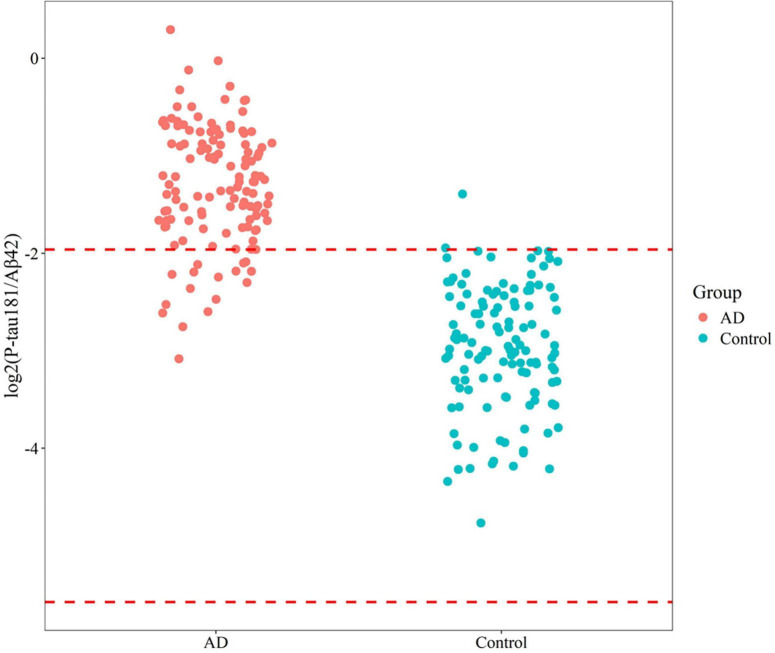
Reference interval distribution of P-tau181/Aβ42 in AD/MCI and control groups based on SIMOA platform.

An independent validation was conducted on a cohort from Xuanwu Hospital, comprising 122 controls (70.0 ± 7.1 years) and 126 AD/MCI patients (69.0 ± 6.3 years). This analysis established a SIMOA-based reference interval for P-tau181/Aβ42, ranging from 0.021 to 0.257. The diagnostic performance of this biomarker exhibited comparable accuracy (92.3%), sensitivity (86.5%), and specificity (98.4%) to that observed with flow cytometry (Figure 5). These findings emphasize the robust diagnostic utility of P-tau181/Aβ42 across both SIMOA and flow cytometry platforms, highlighting its significant potential for clinical applications.

## Discussion

This study established that the plasma P-tau181/Aβ42 ratio, measured via a novel flow cytometric assay, demonstrated 96.2% diagnostic accuracy in discriminating AD patients from age-matched controls when benchmarked against Aβ-PET imaging results. The assay showed comparable diagnostic performance to SIMOA-quantified P-tau217 but weak inter-assay reproducibility. CV in normal controls may reflect biological variability linked to age or pre-pathological Aβ changes. For clinical translation, we recommend averaging duplicate measurements and age-stratifying reference ranges in future studies. This methodological framework extends previous research through three key advancements: (a) establishment of multicenter-derived reference intervals (0–0.109 pg/mL), (b) prospective validation using Aβ-PET-confirmed incident cases, and (c) rigorous cross-platform standardization against the established SIMOA reference method.

Contemporary blood-based Aβ and P-tau quantification necessitates ultrasensitive detection platforms such as SIMOA technology or immunoprecipitation coupled with mass spectrometry (IP-MS) to measure sub-picomolar concentrations (van Dyck et al., 2023). Although these methods demonstrate excellent analytical precision, their substantial operational costs and requirement for specialized infrastructure constrain widespread clinical implementation. In contrast, our optimized flow cytometric assay achieves comparable sensitivity at lower reagent cost, with minimal technical requirements suited for primary care settings. Unlike chemiluminescence or radioimmunoassays, which lack sensitivity for sub-pg/mL analytes, our platform’s robust performance underscores its viability for population-scale screening.

The reference interval (0–0.109) exhibited stability across age groups (*r* = 0.16, *p* = 0.01 for age correlation), critical for minimizing false positives in longitudinal monitoring. We analyzed the consistency of P-tau181/Aβ42 with Aβ pathology results based on Aβ-PET to determine its accuracy. Data analysis showed that P-tau181/Aβ42 based on flow cytometry has good consistency with Aβ-PET diagnostic results, with an accuracy rate of 96.2%, while the accuracy of the SIMOA platform is 92.3%. Notably, the P-tau181/Aβ42 ratio achieved an accuracy of 0.962 for Aβ pathology results based on Aβ-PET. Standalone P-tau181 yielded an accuracy of 95.6%, while the Aβ42/40 ratio had an accuracy of 70.5% (Supplementary Table 3), which indicated that plasma P-tau181/Aβ42 outperformed standalone P-tau181 or Aβ42/40 ratios, aligning with findings that composite biomarkers better reflect cerebral Aβ burden (Fowler et al., 2022). Despite lacking direct CSF comparisons—a study limitation—our Aβ-PET correlations strongly suggest the marker’s utility in detecting early amyloidosis.

While SIMOA P-tau217 remains the non-invasive gold standard (Sims et al., 2023), our flow cytometric P-tau181/Aβ42 assay demonstrates comparable accuracy (88.3% agreement) and superior cost-effectiveness position it for rapid adoption in resource-limited regions. In summary, through multiple comparisons, we have found that the P-tau181/Aβ42 ratio on the flow cytometry platform is an effective tool that can assist in the clinical diagnosis of AD. Although there is still a certain gap between the P-tau181/Aβ42 ratio on the flow cytometry platform and the P-tau217 ratio on the SIMOA platform, and more sample validation is needed, this at least indicates its significant potential in the early diagnosis of AD.

Our findings demonstrate the robust diagnostic performance of P-tau181/Aβ42 in differentiating AD from controls and non-AD dementias, offering a non-invasive, cost-effective alternative for scalable screening in resource-limited settings. For high-stakes clinical decisions, we recommend a stepped diagnostic approach: an initial plasma P-tau181/Aβ42 test followed by confirmatory CSF or amyloid PET imaging for patients over the cutoff. This strategy balances diagnostic confidence with cost-effectiveness, particularly in settings where false-positive or false-negative results could lead to significant clinical or financial consequences.

Three key limitations deserve attention. Firstly, while the cohort is phenotypically rigorous, it needs to be expanded to include diverse populations to ensure the generalizability of the findings. While geographic diversity strengthens external validity, unmeasured confounders (e.g., diet, environmental exposures) may contribute to subtle biomarker variability. Future studies should incorporate granular geographic and lifestyle data to further refine AD biomarker interpretation. Besides, studies should monitor kit batch consistency over extended periods to ensure stable diagnostic cutoffs. Secondly, direct correlations between plasma and CSF P-tau181/Aβ42 were not explored, which prevents definitive conclusions about the coupling of peripheral and central biomarkers. Future studies should incorporate paired plasma, CSF and PET data in asymptomatic populations to validate P-tau181/Aβ42’s performance in preclinical AD detection. Finally, potential modifiers such as renal dysfunction or APOE ε4 dosage effects were not addressed—factors known to influence plasma tau levels (Zhuang and Liu, 2023). Nonetheless, our findings confirm that accessible blood-based assays can alleviate systemic underdiagnosis, a crucial step toward equitable AD care.

## Data Availability

The original contributions presented in this study are included in this article/Supplementary material, further inquiries can be directed to the corresponding authors.
